# Statistical Beamforming for Massive MIMO Systems with Distinct Spatial Correlations

**DOI:** 10.3390/s20216255

**Published:** 2020-11-02

**Authors:** Taehyoung Kim, Sangjoon Park

**Affiliations:** 1 Samsung Research, Samsung Electronics Company Ltd., Seoul 06765, Korea; khotdog86@gmail.com; 2 Department of Electronic Engineering, Kyonggi University, Suwon 16227, Korea

**Keywords:** massive MIMO, statistical beamforming, distinct spatial correlations, partial nulling

## Abstract

In this paper, we propose a novel statistical beamforming (SBF) method called the partial-nulling-based SBF (PN-SBF) to serve a number of users that are undergoing distinct degrees of spatial channel correlations in massive multiple-input multiple-output (MIMO) systems. We consider a massive MIMO system with two user groups. The first group experiences a low spatial channel correlation, whereas the second group has a high spatial channel correlation, which can happen in massive MIMO systems that are based on fifth-generation networks. By analyzing the statistical signal-to-interference-plus-noise ratio, it can be observed that the statistical beamforming vector for the low-correlation group should be designed as the orthogonal complement for the space spanned by the aggregated channel covariance matrices of the high-correlation group. Meanwhile, the spatial degrees of freedom for the high-correlation group should be preserved without cancelling the interference to the low-correlation group. Accordingly, a group-common pre-beamforming matrix is applied to the low-correlation group to cancel the interference to the high-correlation group. In addition, to deal with the intra-group interference in each group, the post-beamforming vector for each group is designed in the manner of maximizing the signal-to-leakage-and-noise ratio, which yields additional performance improvements for the PN-SBF. The simulation results verify that the proposed PN-SBF outperforms the conventional SBF schemes in terms of the ergodic sum rate for the massive MIMO systems with distinct spatial correlations, without the rate ceiling effect in the high signal-to-noise ratio region unlike conventional SBF schemes.

## 1. Introduction

New radio (NR), which is a part of the fifth-generation (5G) standards of the Third Generation Partnership Project (3GPP), has been specified recently and successfully commercialized globally [1]. The 5G NR has been designed to meet a set of requirements that are recommended by the International Telecommunication Union for IMT-2020 [2]. In comparison to the fourth-generation (4G) long-term evolution (LTE), the NR supports faster data rates, lower latency, higher reliability, and new spectrum bands for enabling a wide range of use-cases. This includes enhanced mobile broadband (eMBB), ultra-reliable low-latency communications (URLLC), and massive machine-type communications (mMTC) [3].

From a technical point of view, the 5G NR has been specified with multiple big changes from the 4G LTE [1]. First, NR adopts the orthogonal frequency division multiplexing (OFDM) based waveform with variable subcarrier spacing (SCS) from 15 kHz to 120 kHz. Accordingly, NR can provide services with flexible symbol lengths, which enables the service quality optimization depending on use scenarios and the latency adaptation [3]. Second, NR supports up to 400 MHz bandwidth to meet the tremendous peak data rate requirement of 20 Gbps. For this purpose, a higher frequency range such as the mmWave band from 24.25 GHz to 52.6 GHz has started to be used for 5G services. Third, NR utilizes multi-beam operations to overcome the severe propagation loss that happens in the mmWave band. Multiple high-resolution directional beams are used to provide a sufficient signal quality with long range [4].

Massive multiple-input multiple-output (MIMO) is considered to be one of the key features for the 5G NR. With a number of antennas at the base station (BS), massive MIMO systems can remarkably improve the spectral efficiency by supporting a number of users simultaneously for the given time and frequency resources. In addition, a large number of antenna elements can shape very narrow directional beams to overcome the severe path-loss and blockage in mmWave. Therefore, a number of studies have been investigated to fully utilize the benefits of massive MIMO systems [5,6,7,8,9,10,11,12].

The performance and scalability of massive MIMO systems can be limited due to the several practical factors. Hardware impairment is the one of key factors to degrade the performance of massive MIMO systems [13,14,15,16,17]. Non-ideal hardware such as the non-linear amplifier at the transmitter and receiver causes non-linear distortions to the signals, which can yield a significant performance degradation in massive MIMO systems, for example, incorrect beamforming by non-linear amplifications [13].

On the other aspect, the benefits of massive MIMO systems heavily rely on the availability of the channel state information (CSI) at the BS. For the time division duplex (TDD) systems, the downlink CSI at the BS can be easily obtained from the uplink training due to the reciprocity between the downlink and uplink channels [18]. Since the overhead of the uplink training is proportional to the number of users regardless of the number of BS antennas, acquiring a reliable CSI at the BS with a massive number of antennas requires a reasonable overhead [19]. On the other hand, for frequency division duplex (FDD) systems, downlink training and CSI feedback are necessary because the channel reciprocity is not applicable [20]. Furthermore, downlink training in FDD systems requires tremendous overhead because the amount of overhead is scaled with the number of BS antennas [21]. In addition, after downlink training, each user needs to quantize the estimated downlink channel to transmit a CSI feedback message to the BS, which causes additional channel errors and feedback overheads.

To resolve this fundamental bottleneck of the FDD massive MIMO systems, many concepts and schemes on how to reduce the CSI acquisition overhead have been studied [22,23,24,25,26]. In References [22,23], compressed sensing (CS)-based approaches that exploit the sparsity of massive MIMO channels were investigated to reduce the training overhead. In Reference [24], the CS algorithms were developed to further reduce the pilot overhead by considering the temporal correlation of a massive MIMO channel. In Reference [25], the structured turbo CS algorithm for structured sparse signal recovery was presented to reduce the computational complexity and storage requirement. In addition to the CS-based approaches, in Reference [26], trellis-code-based quantization codebooks were proposed to reduce the training and feedback overhead using the time correlation of the channels.

In spite of the various efforts to overcome the drawbacks of the FDD massive MIMO systems, acquiring the instantaneous CSI with a high accuracy remains a challenge. Meanwhile, in comparison with the instantaneous CSI, the statistical CSI can be acquired more easily and accurately. Consequently, there have been several studies that designed the beamforming vector by exploiting the statistical CSI instead of the instantaneous CSI [27,28,29,30,31,32,33,34]. In Reference [27], the optimal statistical beamforming (SBF) structure for the two-user broadcast channel was presented. This was further extended in Reference [28], in which users were selected with orthogonal principal statistical eigen-directions. In Reference [29], a two-staged beamforming method, termed joint spatial division multiplexing (JSDM), was proposed, where the pre-beamforming matrix was obtained based on zero-forcing (ZF) criterion. In addition, the effective channel with a reduced dimension was estimated and fed back to the BS. In References [30,31], enhanced SBF techniques that applied extra information on top of the statistical CSI were studied. In particular, the angle-of-departure (AoD) and the corresponding large-scale fading coefficients were considered in Reference [30], and the effective channel gain was exploited for the SBF design in Reference [31]. In Reference [32], a joint power allocation and beam selection scheme for unicast and multicast transmissions with the statistical CSI was proposed to maximize the energy efficiency. In Reference [33], the joint SBF design and user scheduling was analyzed by considering the signal-to-leakage-and-noise ratio (SLNR)-based SBF. In Reference [34], an iterative analog-digital multi-user equalizer scheme using limited statistical CSI feedback was proposed for the uplink of wideband millimeter-wave massive MIMO systems.

In this study, a specific network environment in which a number of users experiencing distinct spatial channel correlations need to be served in a multi-user MIMO manner is considered. In the current 5G network, this scenario is already considered for wireless communication services as described below.

- NR supports the transmission of physical control channels for the common control and the user-specific control with different beams. For the common control channel, the wide-beam is transmitted to a number of users in the wide-cell area, in which the users can suffer from rich scattering environments. Meanwhile, for the user-specific control channel, the narrow-beam is transmitted for a certain user with line-of-sight environments. Therefore, the distinct spatial channel correlations can be found for the users with a different control channel [35,36].
- NR supports a wireless backhaul capability between a macro BS and a small BS, which is called integrated access and backhaul [37,38,39]. Since the BSs are expected to be installed at very high locations (e.g., at the top of a tall building), the backhaul channel has a much narrower angular spread (AS) in comparison with the access channel between the BSs and the users [39,40], which creates distinct spatial channel correlations in massive MIMO systems.

Thus, without loss of generality, we can consider a scenario with two user groups for distinct spatial channel correlations: (i) a group with a low spatial channel correlation because of a rich spatial scattering environment, and (ii) a group with a high spatial channel correlation because of the lack of scattering.

Although many studies have been presented for a better SBF design, to the best of the authors’ knowledge, there has been little effort to investigate the SBF scheme that considers the specific 5G NR environment with users experiencing distinct spatial channel correlations. Although the conventional SBF schemes can be directly applicable to the specific scenario, there exist several limitations still remained in the massive MIMO systems with distinct spatial correlations. For example, the ZF-based SBF (ZF-SBF) [29], one of the representative SBF schemes, suffers from the lack of degrees of freedom for nulling multi-user interference as the number of served users increases. Since the ZF constraint is fairly tight, only a part of interferences can be eliminated, and the residual interference can yield the performance degradation. Although this performance degradation can be compensated by the additional parameter optimization, the computational complexity becomes infeasible. Meanwhile, the SLNR-based SBF (SLNR-SBF) [33], another representative SBF scheme, has a benefit of the generation of beamforming vectors from the simple closed-form expression. Further, in contrast to the ZF-BSF, the SLNR-SBF does not require any condition regarding degrees of freedom. However, the SLNR-SBF suffers from the *rate ceiling effect*, that is, the sum rate performance is saturated quickly at high signal-to-noise ratio (SNR) region. Consequently, a more effective SBF structure is necessary to overcome these limitations of the conventional schemes in massive MIMO systems with distinct spatial correlations.

Therefore, we propose a new SBF scheme, termed the partial-nulling-based SBF (PN-SBF) scheme, to maximize the sum rate for serving these two user groups in FDD massive MIMO systems with distinct spatial channel correlations. The PN-SBF is designed to consider the degree of channel correlation for FDD massive MIMO systems when only the statistical CSI is available. From this, the expected statistical signal-to-interference-plus-noise ratio (SINR) is defined and analyzed in terms of the spatial degrees of freedom and the eigenvalues of the channel covariance matrix. Based on this analysis, we demonstrate that the interference from the user group with a low spatial correlation to the user group with a high spatial correlation should be completely eliminated to maximize the sum rate. Consequently, a pre-beamforming matrix for the low-correlation user group is designed as the null space of the aggregated channel covariance matrix for the high-correlation user group. In addition, to handle the multi-user interference within each group, the post-beamforming vectors are designed in the manner of maximizing the SLNR [33,41,42,43]. By doing this, the proposed PN-SBF scheme can obtain a significantly high ergodic sum rate in comparison with the convention SBF schemes for massive MIMO systems with distinct spatial channel correlations, which will be verified throughout the remainder of the paper.

The main contributions of this paper can be summarized as below:

- A new SBF structure is proposed for a specific scenario in which a number of users with distinct spatial channel correlations are served in multi-user MIMO manner. This deployment scenario is currently being considered in the most recent 5G standardization. The proposed SBF scheme is developed for such a network environment so that the degrees of the channel correlation of users are considered for designing beamforming vectors. For that, the proposed SBF has a special structure that is composed of the combination of ZF-SBF and SLNR-SBF.
- The proposed SBF scheme is more efficient and robust compared to the existing SBF schemes in massive MIMO systems with distinct spatial correlations. By combining ZF-based approach and SLNR-based approach together, the proposed SBF structure takes the advantages while overcomes drawbacks of the conventional SBF schemes. As a result, the proposed SBF can be obtained by the simple closed-form expression without additional parameter optimizations and can achieve the robustness to the rate ceiling effect in the high SNR region.

The rest of this paper is organized as follows—Section 2 presents the downlink FDD massive MIMO system model. Section 3 introduces the conventional SBF schemes, and Section 4 presents the proposed PN-SBF scheme in detail. Section 5 provides the simulation results to verify the superiority of the PN-SBF, and Section 6 concludes the paper.

***Notations:*** We use boldface capital letters for the matrices and boldface small letters for the vectors. $X^{T}$, $X^{H}$, $\mathrm{tr}\left(X\right)$, ${∥X∥}_{F}$, and $\mathrm{vec}\left(X\right)$ represent the transpose, Hermitian transpose, trace, Frobenius norm, and the vectorization of a matrix X, respectively. $\mathrm{diag}\left(x_{1},\ldots ,x_{n}\right)$ denotes a diagonal matrix with $x_{1},\ldots ,x_{n}$ on its main diagonal and $I_{N}$ represents an N×N identity matrix. $u_{\max}\left(X\right)$ denotes the dominant eigenvector of a matrix X. Finally, $E\left[\cdot \right]$ denotes the mathematical expectation.

## 2. System Model

We consider a downlink multiuser MIMO system with *M* transmission antennas at the BS and *K* single-antenna users served by the BS. There are two user groups that are classified by the spatial correlation: $U_{L}$ for a set of users with a low spatial correlation and $U_{H}$ for the other set of users with a high spatial correlation. Each user belongs to either $U_{L}$ or $U_{H}$ according to the spatial channel correlation that the user experiences. Therefore, $K=K_{L}+K_{H}$, where $K_{L}=\left|U_{L}\right|$ and $K_{H}=\left|U_{H}\right|$.

The downlink channel between the user *k* and the BS is given by an M×1 complex Gaussian random vector as $h_{k}∼CN\left(0,R_{k}\right)$, where $R_{k}\overset{\Delta }{=}E\left[h_{k}h_{k}^{H}\right]$ denotes the channel covariance. The one-ring scattering model is considered for the channel covariance $R_{k}$ [29], and the element of $R_{k}$ at the *m*th row and *p*th column is given by

(1) $${\left[R_{k}\right]}_{m,p}=\frac{1}{2\Delta_{k}}\int_{-\Delta_{k}}^{\Delta_{k}}e^{jk^{T}\left(\phi +\theta_{k}\right)\left(u_{m}-u_{p}\right)}d\phi .$$

In (1), $\theta_{k}$ and $\Delta_{k}$ are the AoD and AS of user *k*, respectively. $k\left(\phi \right)=-\frac{2\pi }{\lambda }{\left(\cos\left(\phi \right),\sin\left(\phi \right)\right)}^{T}$ is the wave vector with AoD ϕ, λ is the carrier wavelength, and $u_{m}\left(u_{p}\right)\in R^{2}$ are the vectors that indicate the position of the antennas *m* (*p*). It is worthwhile to mention that the degree of the channel correlation depends on $\theta_{k}$ and $\Delta_{k}$. In general, a small $\Delta_{k}$ leads to a high spatial correlation between the antenna elements and the effect of $\theta_{k}$ on the correlation varies depending on the antenna array structure. For example, in the uniform circular array, the degree of the correlation is independent of $\theta_{k}$.

Using the Karhunen-Loeve transform [29], the channel vector can be expressed as

(2) $$h_{k}=U_{k}\Lambda_{k}^{1/2}g_{k},$$

where $g_{k}\in C^{r_{k}\times 1}∼CN\left(0,I_{r_{k}}\right)$, $U_{k}\in C^{M\times r_{k}}$ is a matrix whose columns are the eigenvectors of $R_{k}$, $\Lambda_{k}=\mathrm{diag}\left(\lambda_{k,1},\cdots ,\lambda_{k,r_{k}}\right)$ is a matrix whose elements are non-zero eigenvalues of $R_{k}$ with the *i*th eigenvalue $\lambda_{i}$, and $r_{k}$ is the rank of the channel for user *k*.

Without considering the hardware impairment, the received signal of user *k* is expressed as

(3) $$y_{k}=\sqrt{\rho }\underset{\mathrm{desired}\;\mathrm{signal}}{\underset{︸}{h_{k}^{H}w_{k}x_{k}}}\;+\;\sqrt{\rho }\;\underset{\mathrm{multiuser}\;\mathrm{interference}}{\underset{︸}{\sum_{j\neq k}h_{k}^{H}w_{j}x_{j}}}+z_{k},$$

where $w_{k}$ is an M×1 beamforming vector with ${∥w_{k}∥}^{2}=1$, $x_{k}$ is a data symbol with ${\left|x_{k}\right|}^{2}=1$ for user *k*, ρ is the transmit SNR, and $z_{k}∼CN\left(0,1\right)$ is the normalized complex additive white Gaussian noise. Consequently, the corresponding received SINR of user *k* is given by

(4) $$\begin{array}{cc} {\mathrm{SINR}}_{k} & =\frac{\rho {\left|h_{k}^{H}w_{k}\right|}^{2}}{\rho \sum_{j\neq k}{\left|h_{k}^{H}w_{j}\right|}^{2}+1} \end{array}$$

(5) $$\begin{array}{cc} & =\frac{\rho w_{k}^{H}U_{k}\Lambda_{k}^{1/2}g_{k}g_{k}^{H}\Lambda_{k}^{1/2}U_{k}^{H}w_{k}}{\rho \sum_{j\neq k}w_{j}^{H}U_{k}\Lambda_{k}^{1/2}g_{k}g_{k}^{H}\Lambda_{k}^{1/2}U_{k}^{H}w_{j}+1}. \end{array}$$

Therefore, the achievable ergodic sum rate can be expressed as

(6) $$\begin{array}{cc} R_{\mathrm{sum}} & \overset{\Delta }{=}E\left[\sum_{k=1}^{K}R_{k}\right]\\ & =E\left[\sum_{k=1}^{K}\log_{2}\left(1+{\mathrm{SINR}}_{k}\right)\right]. \end{array}$$

## 3. Conventional Statistical Beamforming Schemes

In general, designing an SBF scheme that directly maximizes the ergodic sum rate is very challenging because the achievable rate in (6) includes the complicated functions of the channel covariance and the beamforming vectors [33]. Accordingly, many existing studies focus on the design of low complexity SBF schemes [27,28,29,30,31,32,33]. Among them, we briefly present two representative SBF schemes: the ZF-SBF [29] and the SLNR-based SBF (SLNR-SBF) [33].

### 3.1. Zero-Forcing-Based Statistical Beamforming

ZF-SBF is a special case of the JSDM in Reference [29], in which each user group includes only a single user and a single data stream is transmitted to each user. For ZF-SBF, the criterion for choosing the beamforming vector $w_{k}$ is based on the following ZF condition.

(7) $$U_{j}^{H}w_{k}=0,\;\forall j\neq k.$$

The ZF-SBF that satisfies the condition in (7) can achieve a fine performance since the multiuser interference is completely cancelled. However, to find the solutions $w_{k}$ that satisfy (7) for all the *k* values, the following constraint needs to be satisfied.

(8) $$M>\sum_{j\neq k}r_{j},\;\forall k.$$

Since the number of served users and the channel rank for each user should be sufficiently small, the constraint (8) is fairly tight, even when *M* is very large. Accordingly, when the constraint (8) cannot be satisfied, the beamforming vector can be designed in the manner of the *approximated* ZF approach [29]. That is, by choosing $r_{k}^{*}$ dominant eigenmodes of $U_{k}$ with the constraint of $M>\sum_{j\neq k}r_{j}^{*}$∀k, we can obtain the beamforming vector that satisfies the following condition.

(9) $${\left(U_{j}^{*}\right)}^{H}w_{k}=0,\;\forall j\neq k.$$

$U_{k}=\left[U_{k}^{*},U_{k}^{\circ }\right]$, $U_{k}^{*}$ is an $M\times r_{k}^{*}$ matrix that collects $r_{k}^{*}$ dominant eigenmodes, and $U_{k}^{\circ }$ is an $M\times \left(r_{k}-r_{k}^{*}\right)$ matrix that contains $\left(r_{k}-r_{k}^{*}\right)$ non-dominant eigenmodes. To satisfy the condition in (9), the beamforming vector should be in the null space of $\mathrm{Span}({\tilde{U}}_{k})$, where ${\tilde{U}}_{k}$ is defined as

(10) $${\tilde{U}}_{k}=\left[U_{1}^{*}\cdots U_{k-1}^{*},U_{k+1}^{*}\cdots U_{K}^{*}\right].$$

Let $E_{k}=\left[E_{k}^{\left(1\right)},E_{k}^{\left(0\right)}\right]$ denote a matrix corresponding to the left eigenvectors of ${\tilde{U}}_{k}$ that is obtained by singular value decomposition (SVD). $E_{k}^{\left(0\right)}$ is an $M\times n_{k}$ matrix with $n_{k}=M-\sum_{j\neq k}r_{j}^{*}$, which corresponds to the null space of $\mathrm{Span}({\tilde{U}}_{k})$. Let ${\hat{h}}_{k}\overset{\Delta }{=}{\left(E_{k}^{\left(0\right)}\right)}^{H}h_{k}$ be the effective channel that is obtained by projecting $h_{k}$ onto $E_{k}^{\left(0\right)}$. Subsequently, the covariance matrix of the effective channel ${\hat{R}}_{k}\overset{\Delta }{=}E\left[{\hat{h}}_{k}{\hat{h}}_{k}^{H}\right]$ can be written as

(11) $${\hat{R}}_{k}={\left(E_{k}^{\left(0\right)}\right)}^{H}U_{k}\Lambda_{k}U_{k}^{H}E_{k}^{\left(0\right)}=V_{k}\Phi_{k}V_{k}^{H},$$

where $\Phi_{k}\left(=\mathrm{diag}\left({\hat{\lambda }}_{k,1},\ldots ,{\hat{\lambda }}_{k,{\hat{r}}_{k}}\right)\right)$ and $V_{k}$ consist of ordered eigenvalues and eigenmodes of ${\hat{R}}_{k}$, respectively, and ${\hat{r}}_{k}$ is the rank of ${\hat{R}}_{k}$. Let $v_{k}$ be the first column vector of $V_{k}$, which corresponds to the largest eigenvalue. Subsequently, the ZF-SBF vector for user *k* is given by

(12) $$w_{k}=E_{k}^{\left(0\right)}v_{k}.$$

Note that it is necessary to find the optimal set of design parameters ${\left\{r_{k,\mathrm{opt}}^{*}\right\}}_{k=1}^{K}$ for maximizing the ergodic sum rate. However, finding the optimal set of parameters requires an exhaustive search, which has an infeasible computational complexity. For simplicity, it is assumed that the dominant eigenmodes of all the users are equally selected with satisfying the constraint (8) as $r_{k}^{*}=\min\left(M/\left(K-1\right),r_{k}\right),\forall k$.

### 3.2. Signal-to-Leakage-and-Noise Ratio Based Statistical Beamforming

For the SLNR-SBF, the SLNR metric of user *k* can be defined as [42]

(13) $${\mathrm{SLNR}}_{k}=\frac{\rho {\left|h_{k}^{H}w_{k}\right|}^{2}}{\rho \sum_{j\neq k}{\left|h_{j}^{H}w_{k}\right|}^{2}+1},$$

where ${\left|h_{j}^{H}w_{k}\right|}^{2}$ in the denominator represents the power leaked from user *k* to user *j*. Considering the availability of only the statistical CSI at the BS, the *statistical* SLNR derived from Mullen’s inequality in Reference [28] is employed for the design of the SLNR-SBF [33]. The statistical SLNR for user *k* is defined as

(14) $$\begin{array}{c} {\mathrm{SLNR}}_{k}^{\mathrm{avg}}\overset{\Delta }{=}\frac{E\left[\rho {\left|h_{k}^{H}w_{k}\right|}^{2}\right]}{E\left[\rho \sum_{j\neq k}{\left|h_{j}^{H}w_{k}\right|}^{2}+1\right]}\\ =\frac{\rho w_{k}^{H}R_{k}w_{k}}{\rho w_{k}^{H}\left(\sum_{j\neq k}R_{j}\right)w_{k}+1}. \end{array}$$

By applying the Rayleigh-Ritz quotient theorem [41], the beamforming vector that maximizes the statistical SLNR can be derived as

(15) $$w_{k}=u_{\max}\left({\left(\rho^{-1}I_{M}+\sum_{j\neq k}R_{j}\right)}^{-1}R_{k}\right).$$

Note that maximizing the SLNR does not necessarily maximize the ergodic sum rate. Nevertheless, in Reference [42] and the references therein, it is demonstrated that the SLNR-SBF can achieve a fine ergodic sum rate.

## 4. Proposed Partial-Nulling-Based Statistical Beamforming

In this section, the proposed PN-SBF scheme that is designed for supporting a number of users with distinct spatial correlations is described. The PN-SBF is designed to satisfy the following two conditions: (i) the robustness to rate ceiling effect and (ii) the formulation from the closed-form expression without additional parameter optimization. To satisfy the first condition (i), ZF-based approach is necessary since the rate ceiling effect occurs due to the residual multi-user interference. We exploit the fact that the ZF condition in (8) can be satisfied more easily as the rank of channel becomes smaller. That is, ZF-based approach can be efficiently used for nulling interference from low-correlation users to high-correlation users. As a result, a ZF-based SBF structure is employed to handle the inter-group interference between two user groups. For the second condition (ii), SLNR-based approach is the most relevant solution since it does not require any dimension condition and has a closed-form structure. Thus, the SLNR-based SBF is applied to mitigate the intra-group interference in each group. Consequently, the PN-SBF can be formulated by a combination of the ZF-SBF and SLNR-SBF principles. In other words, the inter-group interference is mitigated by the pre-beamforming matrix that is designed in the manner of the ZF. Meanwhile, the intra-group interference is handled by the post-beamforming vector that maximizes the SLNR metric. This design principle will be explained in detail throughout the remainder of this section.

First, the statistical SINR of each user is analyzed. The statistical SINR can be defined as

(16) $$\begin{array}{cc} {\mathrm{SINR}}_{k}^{\mathrm{avg}} & \overset{\Delta }{=}\frac{E\left[\rho {\left|h_{k}^{H}w_{k}\right|}^{2}\right]}{E\left[\rho \sum_{j\neq k}{\left|h_{k}^{H}w_{j}\right|}^{2}+1\right]} \end{array}$$

(17) $$\begin{array}{cc} & =\frac{\rho w_{k}^{H}U_{k}\Lambda_{k}^{1/2}E\left[g_{k}g_{k}^{H}\right]\Lambda_{k}^{1/2}U_{k}^{H}w_{k}}{\rho \sum_{j\neq k}w_{j}^{H}U_{k}\Lambda_{k}^{1/2}E\left[g_{k}g_{k}^{H}\right]\Lambda_{k}^{1/2}U_{k}^{H}w_{j}+1} \end{array}$$

(18) $$\begin{array}{cc} & =\frac{\rho w_{k}^{H}U_{k}\Lambda_{k}U_{k}^{H}w_{k}}{\rho \sum_{j\neq k}w_{j}^{H}U_{k}\Lambda_{k}^{1/2}U_{k}^{H}w_{j}+1}. \end{array}$$

Assuming that ZF-SBF is employed, the statistical SINR can be re-formulated by substituting (12) into (18) as

(19) $$\begin{array}{cc} {\mathrm{SINR}}_{k}^{\mathrm{avg}} & =\frac{\rho v_{k}^{H}{\left(E_{k}^{\left(0\right)}\right)}^{H}U_{k}\Lambda_{k}U_{k}^{H}E_{k}^{\left(0\right)}v_{k}}{\rho \sum_{j\neq k}w_{j}^{H}U_{k}^{\circ }\Lambda_{k}^{\circ }{\left(U_{k}^{\circ }\right)}^{H}w_{j}+1} \end{array}$$

(20) $$\begin{array}{cc} & \overset{\left(a\right)}{=}\frac{\rho {\hat{\lambda }}_{k,1}}{\rho \sum_{j\neq k}w_{j}^{H}U_{k}^{\circ }\Lambda_{k}^{\circ }{\left(U_{k}^{\circ }\right)}^{H}w_{j}+1}, \end{array}$$

where (a) is derived from the fact that $v_{k}$ is the dominant eigenvector that corresponds to the largest eigenvalue ${\hat{\lambda }}_{k,1}$ of ${\hat{R}}_{k}$ defined in (11). $\Lambda_{k}^{\circ }=\mathrm{diag}\left(\lambda_{k,r_{k}^{*}+1},\lambda_{k,r_{k}^{*}+2},\cdots ,\lambda_{k,r_{k}}\right)$ is a matrix that contains $\left(r_{k}-r_{k}^{*}\right)$ non-dominant eigenvalues of $R_{k}$.

From the numerator in (20), it is observed that the quality of the desired signal term ${\hat{\lambda }}_{k,1}$ depends on (i) $n_{k}=M-\sum_{j\neq k}r_{j}^{*}$ and (ii) $U_{k}^{H}E_{k}^{\left(0\right)}$. $n_{k}$ corresponds to the remaining spatial degrees of freedom of user *k* after sacrificing the degrees of freedom to cancel the interference from user *k* to the other users. That is, as $n_{k}$ increases, the degrees of freedom for user *k* is designed to enhance its own signal quality rather than mitigate the interference. Therefore, we can expect an increase in ${\hat{\lambda }}_{k,1}$ with $n_{k}$. Meanwhile, $U_{k}^{H}E_{k}^{\left(0\right)}$ corresponds to the orthogonality between $\mathrm{Span}\left(U_{k}^{*}\right)$ and $\mathrm{Span}\left(U_{j}^{*}:j\neq k\right)$. Thus, if $U_{k}^{*}$ is exactly on the $\mathrm{Spa}n^{⊥}\left(U_{j}^{*}:j\neq k\right)$, that is, $E_{k}^{\left(0\right)}=U_{k}^{*}$, ${\hat{\lambda }}_{k,1}$ can be maximized. Therefore, when $n_{k}=M$ and $E_{k}^{\left(0\right)}=U_{k}$, for example, an extreme case, the desired signal term ${\hat{\lambda }}_{k,1}$ is maximized as ${\hat{\lambda }}_{k,1}=\lambda_{k,1}$.

On the other hand, the denominator in (20) shows that the multiuser interference term depends on $r_{k}^{*}$ and $\Lambda_{k}^{\circ }$. $r_{k}^{*}$ corresponds to the number of dominant eigenmodes that are cancelled by the beamforming vectors of the other users. In addition, $\mathrm{tr}\left\{\Lambda_{k}^{\circ }\right\}$ corresponds to the quantity of the residual interference from the $\left(r_{k}-r_{k}^{*}\right)$ weakest eigenmodes. Therefore, to minimize the multiuser interference, a large $r_{k}^{*}$ and a small $\mathrm{tr}\left\{\Lambda_{k}^{\circ }\right\}$ are required.

Consequently, to maximize the statistical SINR, the parameters ${\left\{r_{k}^{*}\right\}}_{k=1}^{K}$ should be jointly optimized by considering the covariance matrices for all of the users, that is, ${\left\{R_{k}\right\}}_{k=1}^{K}$, but the direct optimization of this problem is an infeasible task. Thus, to simplify the optimization problem, we exploit the fact that $R_{k}$ is independent of $E_{k}^{\left(0\right)}$ and $U_{k}^{\circ }\Lambda_{k}^{\circ }{\left(U_{k}^{\circ }\right)}^{H}$ is independent of ${\left\{w_{j}\right\}}_{j\neq k}$. Using these independencies, we can consider a new metric, the *expected* statistical SINR, which is defined as

(21) $${\bar{\mathrm{SINR}}}_{k}^{\mathrm{avg}}\overset{\Delta }{=}\frac{\rho E_{E^{\left(0\right)}}\left[v_{k}^{H}{\left(E_{k}^{\left(0\right)}\right)}^{H}U_{k}\Lambda_{k}U_{k}^{H}E_{k}^{\left(0\right)}v_{k}\right]}{\rho E_{w}\left[\sum_{j\neq k}w_{j}^{H}U_{k}^{\circ }\Lambda_{k}^{\circ }{\left(U_{k}^{\circ }\right)}^{H}w_{j}\right]+1},$$

where $E_{E}\left[\cdot \right]$ and $E_{w}\left[\cdot \right]$ represent the expectation operations in terms of $E_{k}^{\left(0\right)}$ and ${\left\{w_{j}\right\}}_{j\neq k}$, respectively. Note that $E_{k}^{\left(0\right)}$ and ${\left\{w_{j}\right\}}_{j\neq k}$ are regarded as random variables in (21). Subsequently, we have the following lemma for the expected statistical SINR.

**Lemma** **1.** *The expected statistical SINR in (21) can be approximated as follows.*

(22) $${\bar{\mathrm{SINR}}}_{k}^{\mathrm{avg}}\approx {\bar{\mathrm{SINR}}}_{k}^{\mathrm{app}}=\frac{\rho \frac{M-\sum_{j\neq k}r_{j}^{*}}{M}\lambda_{k,1}}{\rho \frac{K-1}{M}\sum_{i=r_{k}^{*}+1}^{r_{k}}\lambda_{k,i}+1}.$$

**Proof.**  See Appendix A. □

Therefore, when using the approximation in (22) of Lemma 1, the optimization problem to find ${\left\{r_{k,\mathrm{opt}}^{*}\right\}}_{k=1}^{K}$ can be simplified because only $R_{k}$ needs to be considered for the expected statistical SINR instead of ${\left\{{r_{k}}^{*}\right\}}_{k=1}^{K}$ for the statistical SINR. Unfortunately, the optimization problem to maximize the ergodic sum rate using the approximated SINR in (22) is still a mixed integer nonlinear programming (MINLP) problem and obtaining the optimal solution as a closed-form expression is also still infeasible. Thus, as an alternative approach, we consider an upper bound of (22) as

(23) $${\bar{\mathrm{SINR}}}_{k}^{\mathrm{app}}\leq {\bar{\mathrm{SINR}}}_{k}^{\mathrm{upper}}=\frac{\rho \frac{M-\sum_{j\neq k}r_{j}^{*}}{M}\lambda_{k,1}}{\rho \frac{K-1}{M}\left(M-r_{k}^{*}\right)\lambda_{k,r_{k}}+1}.$$

The upper bound in (23) is derived from $\sum_{i=r_{k}^{*}+1}^{r_{k}}\lambda_{k,i}\geq \left(M-r_{k}^{*}\right)\lambda_{k,r_{k}}$ since $\lambda_{k,r_{k}}$ is the minimum eigenvalue. To get an insight for how to design the statistical beamforming vectors for two user groups with distinct spatial correlations, we first consider a simpler problem that handles a two-user case. That is, we modeled the two user groups according to the spatial correlation as two users with distinct spatial correlations. Accordingly, the closed-form expression of the optimal parameters for the two-user case ${\left\{r_{k,\mathrm{opt}}^{*}\right\}}_{k=1}^{2}$ that maximizes the upper bound of the ergodic sum rate can be derived, which is demonstrated in the following theorem.

**Theorem** **1.** Let us consider the two-user case. $R_{k}$ and $R_{l}$ are the covariance matrices for users k and l, respectively. At the high ρ regime, the optimal parameters $\left(r_{k,\mathrm{opt}}^{*},r_{l,\mathrm{opt}}^{*}\right)$ maximize the upper bound of the ergodic sum rate, which are given by

(24) $$\left(r_{k,\mathrm{opt}}^{*},r_{l,\mathrm{opt}}^{*}\right)=\left\{\begin{array}{cc} \left(r_{k},0\right) & \kappa \left(R_{k}\right)\geq \kappa \left(R_{l}\right)\\ \left(0,r_{l}\right) & \kappa \left(R_{k}\right)<\kappa \left(R_{l}\right) \end{array}\right.,$$

*where $\kappa \left(X\right)$ denotes the condition number of the matrix X.*

**Proof.**  See Appendix B. □

Theorem 1 provides an important insight to design the beamforming vector for massive MIMO systems with distinct spatial correlations. From this, consider the physical meaning of the condition number $\kappa \left(R_{k}\right)$ of user *k*. For the highly correlated channel, the direction of the channel is heavily dominated by the dominant eigenmode, which leads to a large condition number, that is, large $\lambda_{k,1}$ and small $\lambda_{k,r_{k}}$. Accordingly, Theorem 1 implies that consuming the spatial degrees of freedom to mitigate the interference to the other user is not necessary to design a beamforming vector for a user with a high spatial correlation. On the other hand, to maximize the ergodic sum rate, the beamforming vector of a user with a low spatial correlation should be designed to perfectly cancel the interference to a user with a high spatial correlation.

Therefore, by applying Theorem 1 from a two-user case to the two-group case (i.e., the user group with a high spatial correlation $U_{H}$ and the user group with a low spatial correlation $U_{L}$), the system can efficiently choose the appropriate ${\left\{r_{k}^{*}\right\}}_{k=1}^{K}$. This is achieved by applying the degrees of the channel correlations for the users without a complicated optimization task or an exhaustive search. From this, the proposed PN-SBF first designs a beamforming matrix in the manner of ZF. Let ${\tilde{U}}_{H}\overset{\Delta }{=}\left\{U_{i}:i\in U_{H}\right\}$ denote the aggregated covariance matrix that collects the covariance matrices of the users in $U_{H}$, and let $E=\left[E^{\left(1\right)},E^{\left(0\right)}\right]$ denote an $M\times \sum_{i\in U_{H}}r_{i}$ matrix of left eigenvectors of ${\tilde{U}}_{H}$. Subsequently, to completely cancel the interference from $U_{L}$ to $U_{H}$, the beamforming matrix C can be designed as

(25) $$C=E^{\left(0\right)},$$

where $E^{\left(0\right)}$ is an $M\times n_{L}$ matrix that corresponds to the null space of ${\tilde{U}}_{H}$ and $n_{L}=M-\sum_{j\in U_{H}}r_{j}$. Therefore, by performing the partial nulling with C, the inter-group interference from $U_{L}$ to $U_{H}$ can be completely eliminated in the proposed PN-SBF. Note that C should be commonly used for every user in $U_{L}$, whereas the users in $U_{H}$ do not need C.

Although C can eliminate the inter-group interference from $U_{L}$ to $U_{H}$, the intra-group interference from the user in the same group still exists. Therefore, to deal with the intra-group interference without consuming additional spatial degrees of freedom, the proposed PN-SNF further uses the additional beamforming vectors to maximize the SLNR metric of the users in each group. When considering C as the pre-beamforming matrix, the post-beamforming vector is jointly applied with C to determine the overall beamforming vector $w_{k}$ for each user. Therefore, $w_{k}$ can be written as

(26) $$w_{k}=\left\{\begin{array}{c} v_{k},\;\mathrm{for}\;k\in U_{H}\\ Cv_{k},\;\mathrm{for}\;k\in U_{L} \end{array}\right.,$$

where the pre-beamforming matrix C is commonly applied to all of the users in $U_{L}$ to eliminate the inter-group interference to the users in $U_{H}$. Meanwhile, C is not applied to the user in $U_{H}$ to use the degrees of freedom.

Next, the post-beamforming vector and the overall beamforming vector for the user *h* in $U_{H}$ are derived. By applying (25) and (26), the received signal in (3) for user *h* can be rewritten as

(27) $$\begin{array}{cc} y_{h} & =\sqrt{\rho }h_{h}^{H}w_{h}x_{h}+\sqrt{\rho }\sum_{j\neq h,j\in U_{H}}h_{h}^{H}w_{j}x_{j}+\sqrt{\rho }\sum_{j\in U_{L}}h_{h}^{H}E^{\left(0\right)}v_{j}x_{j}+z_{h}\\ & =\sqrt{\rho }h_{h}^{H}w_{h}x_{h}+\sqrt{\rho }\sum_{j\neq h,j\in U_{H}}h_{h}^{H}w_{j}x_{j}+z_{h}. \end{array}$$

Let $v_{h}$ denote the M×1 post-beamforming vector for user *h*. As shown in (27), the inter-group interference from $U_{L}$ is completely eliminated by the pre-beamforming matrix C. Therefore, it is sufficient to consider the interference among the users in $U_{H}$ to obtain $v_{h}$. Thus, using the SLNR-based SBF structure in (15), $v_{h}$ can be written as

(28) $$v_{h}=u_{\max}\left({\left(\rho^{-1}I_{M}+\sum_{j\neq h,j\in U_{H}}R_{j}\right)}^{-1}R_{h}\right),\;\mathrm{for}\;h\in U_{H},$$

which is equivalent to the overall beamforming vector $w_{h}\left(=v_{h}\right)$.

Finally, the post-beamforming vector and the overall beamforming vector for user *l* in $U_{L}$ are derived. The received signal in (3) for user *l* can be rewritten by applying (26) as

(29) $$y_{l}=\sqrt{\rho }h_{l}^{H}Cv_{l}x_{l}+\sqrt{\rho }\sum_{j\neq l,j\in U_{L}}h_{l}^{H}Cv_{j}x_{j}+\sqrt{\rho }\sum_{j\in U_{H}}h_{l}^{H}w_{j}x_{j}+z_{l},$$

where $v_{l}$ is the $n_{L}\times 1$ post-beamforming vector for user *l*. By applying $v_{h}$ in (28), the interference power from $U_{H}$ can be estimated as

(30) $${\hat{\sigma }}_{j}^{2}=E\left\{w_{j}^{H}h_{l}h_{l}^{H}w_{j}\right\}=w_{j}^{H}R_{l}w_{j},\;\;j\in U_{H}.$$

Therefore, when using the SLNR-based SBF structure in (15), $v_{l}$ can be derived as

(31) $$v_{l}=u_{\max}\left({\left(\left(\rho^{-1}+\sum_{j\in U_{H}}{\hat{\sigma }}_{j}^{2}\right)I_{n_{L}}+\sum_{j\neq l,j\in U_{L}}{\hat{R}}_{j}\right)}^{-1}{\hat{R}}_{l}\right),\;\mathrm{for}\;l\in U_{L},$$

where ${\hat{R}}_{l}$ is the effective channel covariance matrix after applying the pre-beamforming matrix C, that is,

(32) $${\hat{R}}_{l}=C^{H}R_{l}C.$$

Thus, the overall beamforming vector $w_{l}$ for user *l* is obtained as $Cv_{l}$ when using (26).

In summary, the proposed PN-SBF is formulated by combining the ZF-SBF and SLNR-SBF principles. For the distinct spatial correlation scenario, the inter-group interference from $U_{L}$ to $U_{H}$ is mitigated by the pre-beamforming matrix that is designed in the manner of the ZF. Meanwhile, the intra-group interference is handled by the post-beamforming vector for maximizing the SLNR metric. By doing this, the proposed PN-SBF overcomes the drawbacks that are observed in the conventional SBF schemes, which are described below.

- For the ZF-SBF, it is required to optimize a set of parameters that correspond to the number of dominant eigenmodes that are selected. This optimization task is infeasible because of the enormous computational complexity. Without these optimizations, the performance of the ZF-SBF can be significantly degraded. By contrast, the PN-SBF has a closed-form structure that does not require additional parameter optimization.
- For both ZF-SBF and SLNR-SBF, the multiuser interference cannot be completely eliminated, which can cause the rate ceiling effect in the high SNR region [44]. By contrast, the PN-SBF can obtain more robustness to the rate ceiling effect by employing the partial nulling that is based on the ZF approach to cancel the inter-group interference.

## 5. Simulation Results

This section evaluates the performance of the SBF schemes. We assume that the BS is equipped with a uniform circular array with *M* antennas that are equally spaced on a circle of radius λD with $D=\frac{0.5}{\sqrt{{\left(1-\cos\left(2\pi /M\right)\right)}^{2}+\sin{\left(2\pi /M\right)}^{2}}}$. In addition, the minimum distance between the antennas is equal to λ/2 [29]. The AoDs of the users, that is, $\theta_{k}$, ∀k, are uniformly distributed on $\left[-180^{\circ },\;180^{\circ }\right]$. The ASs for the users in $U_{H}$ and $U_{L}$ are randomly generated from $\left[\Delta_{H}-\delta_{H},\;\Delta_{H}+\delta_{H}\right]$ and $\left[\Delta_{L}-\delta_{L},\;\Delta_{L}+\delta_{L}\right]$, respectively, where $\delta_{H}=\Delta_{H}/2$ and $\delta_{L}=\Delta_{L}/3$. For the ZF-SBF, the number of dominant eigenmodes for all of the users is $r_{k}^{*}=min\left(M/\left(K-1\right),r_{k}\right)$, that is, the ZF condition of $M>\sum_{j\neq k}r_{j}^{*}$ can be always ensured for the ZF-SBF.

In addition to the proposed PN-SBF, ZF-SBF, and SLNR-SBF, the matched-filter based SBF (MF-SBF), one of the representative techniques in massive MIMO systems [5,6,7,8], is considered as well. Typically, compared to other linear beamforming techniques, MF-based approach has the simplest structure and achieves a lower bound of the performance. Despite such limitations, MF-based approach is optimal for non-correlated massive MIMO system with instantaneous CSI [5]. Therefore, the performance of MF-SBF is evaluated in this section in order to figure out how much sum rate can be achieved by MF-based approach in massive MIMO systems with spatial correlations and statistical CSI. For MF-SBF, the beamforming vector $w_{k}$ is selected as the first eigenmode that corresponds to the largest eigenvalue of $R_{k}$.

**Figure 1:**
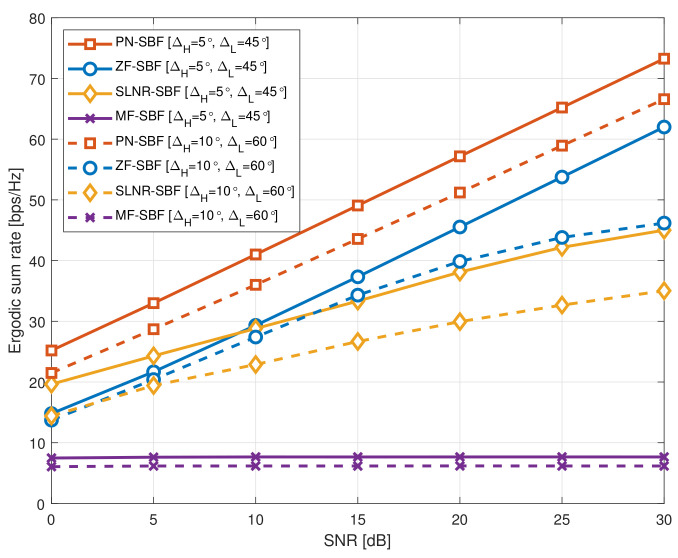
Ergodic sum rate of the statistical beamforming (SBF) schemes according to the spatial correlation, where M=128, $K_{H}=5$, and $K_{L}=15$.

Figure 1 shows the ergodic sum rate of the SBF schemes according to the spatial correlation, where M=128, $K_{H}=5$, and $K_{L}=15$. It is observed that the proposed PN-SBF outperforms the other SBF schemes regardless of the SNR. To be specific, for a high spatial correlation ($\Delta_{H}=5^{\circ }$ and $\Delta_{H}=45^{\circ }$), the rate ceiling effect in the high SNR region is not observed for the PN-SBF and ZF-SBF; however, it is observed for the SLNR-SBF. This is because a part of the multi-user interference is suppressed to zero by the ZF-based design principle of the PN-SBF and ZF-SBF. However, for a low spatial correlation ($\Delta_{H}=10^{\circ }$ and $\Delta_{H}=60^{\circ }$), the ZF-SBF begins to show the rate ceiling effect in the high SNR region. This is because the multi-user interference cannot be eliminated properly, with the ZF-SBF under the low spatial correlation environment. In the ZF-SBF, only a part of the eigenmodes that do not exceed the degrees of freedom *M* can be selected. Therefore, a part of the multi-user interference that was intended to be eliminated still remains. By contrast, for the PN-SBF, the inter-group interference from $U_{L}$ to $U_{H}$ is removed by the ZF-based design, and the intra-group interference is suppressed by the SLNR-based design. Consequently, for both low and high spatial correlations, the PN-SBF does not experience the rate ceiling effect. From this, the proposed PN-SBF outperforms the conventional SBF schemes regardless of the SNR and the spatial correlation. Meanwhile, the optimality of the MF-based beamforming with the instantaneous CSI was verified [5,6,7]. However, the MF-SBF does not consider multi-user interference for the beamforming design, and therefore the optimality of the MF-based beamforming with the instantaneous CSI becomes strictly limited when only a statistical CSI is available at the BS. Consequently, the MF-SBF shows a significantly degraded ergodic sum rate in comparison with the other SBF schemes.

**Figure 2:**
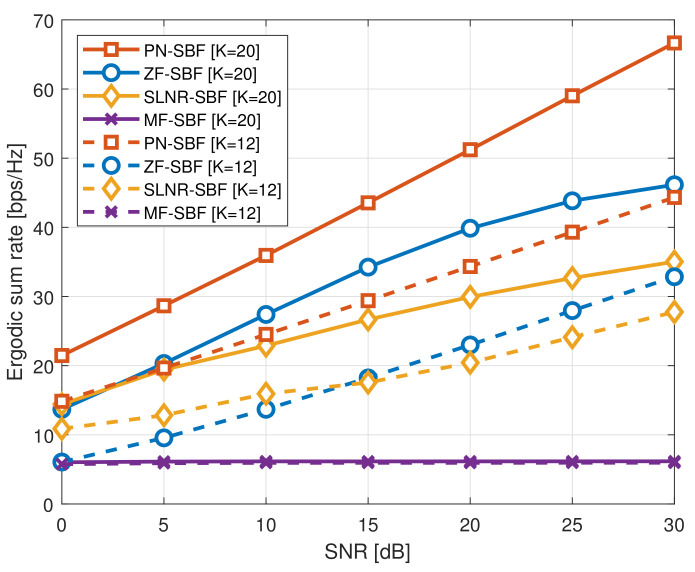
Ergodic sum rate of the SBF schemes, where M=128, $K_{H}=K/4$, $K_{L}=3K/4$, $\Delta_{H}=10^{\circ }$, and $\Delta_{L}=60^{\circ }$.

Figure 2 illustrates the ergodic sum rate of the SBF schemes according to the number of served users *K*, where M=128, $\Delta_{H}=10^{\circ }$, $\Delta_{L}=60^{\circ }$, $K_{H}=K/4$, and $K_{L}=3K/4$. Similar to the results in Figure 1, the proposed PN-SBF achieves better ergodic sum rates than the conventional SBF schemes for a given SNR and *K*. In particular, when K=12, for example, a small number of served users, no rate ceiling effect is observed for ZF-SBF and SLNR-SBF because there are not enough degrees of freedom per user; however, they suffer from the rate ceiling effect when K=20, for example, a large number of served users. On the other hand, the rate ceiling effect is not observed for the PN-SBF regardless of *K*, and the proposed PN-SBF obtains a higher ergodic sum rate than the conventional SBF schemes.

**Figure 3:**
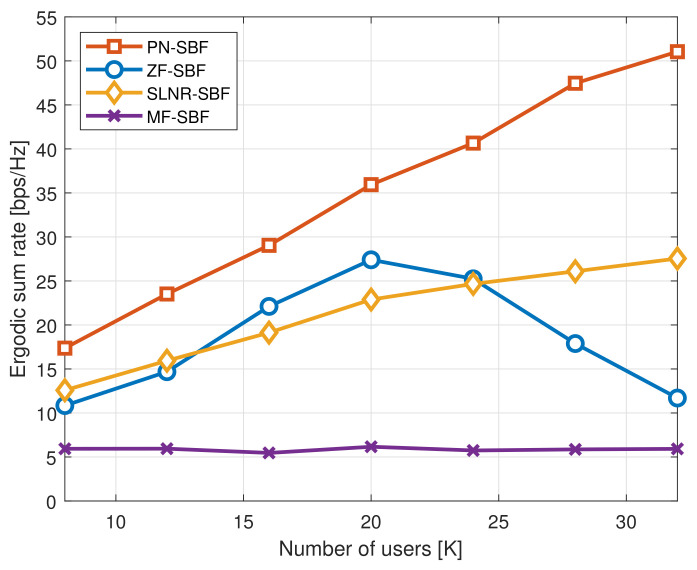
Ergodic sum rate as a function of *K*, where M=128, ρ=10 dB, $\Delta_{H}=10^{\circ }$, $\Delta_{L}=60^{\circ }$, $K_{H}=K/4$, and $K_{H}=3K/4$.

**Figure 4:**
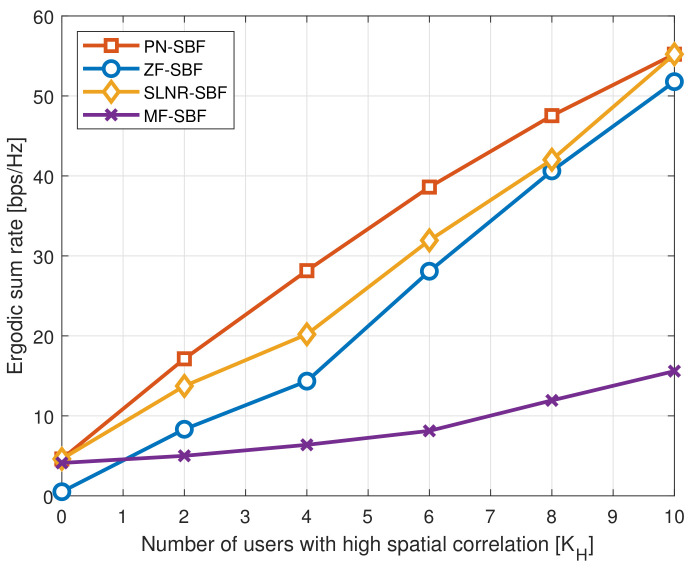
Ergodic sum rate as a function of $K_{H}$, where M=128, ρ=10 dB, $\Delta_{H}=10^{\circ }$, $\Delta_{L}=60^{\circ }$, K=10, and $K_{L}=K-K_{H}$.

To verify the impact of the number of users on the performance more precisely, Figure 3 and Figure 4 show the ergodic sum rates as a function of *K* and $K_{H}$, respectively, where M=128, ρ=10 dB, $\Delta_{H}=10^{\circ }$, and $\Delta_{L}=60^{\circ }$. Furthermore, $K_{H}=K/4$ and $K_{L}=3K/4$ in Figure 3, and K=10 and $K_{L}=K-K_{H}$ in Figure 4.

Figure 3 shows that the ergodic sum rates of the PN-SBF and SLNR-SBF increase linearly to *K*. On the other hand, the ergodic sum rate of the ZF-SBF increases with *K* for the small *K* regime, and it decreases with *K* for the large *K* regime. This is because the degrees of freedom per user that can be consumed for the interference cancellation is reduced as *K* increases; therefore, the multi-user interference cannot be properly removed for the ZF-SBF [45]. Meanwhile, the SLNR-SBF shows a consistent performance improvement with *K*. Accordingly, the SLNR-SBF begins to outperform the ZF-SBF for a large *K*. This implies that the SLNR-based beamforming design is appropriate to serve a large number of users *K*. For the PN-SBF, because only a part of the interference (i.e., inter-group interference) is removed by the ZF-based design, the proposed PN-SBF shows robustness due to the lack of the degrees of freedom in comparison with the ZF-SBF. Furthermore, in addition to the ZF-based design for the inter-group interference, the SLNR-based design for the intra-group interference is applied to the PN-SBF. Therefore, the proposed PN-SBF shows a significantly improved ergodic sum rate in comparison with the other SBF schemes regardless of *K*.

In Figure 4, it is demonstrated that the ergodic sum rate for all of the SBF schemes increases with $K_{H}$ because SBF can operate accurately as the spatial channel correlation of the users becomes high. Therefore, even MF-SBF shows a performance improvement for a larger $K_{H}$. Meanwhile, for the extreme cases of (i) no high-correlation users ($K_{H}=0$) and (ii) no low-correlation users ($K_{H}=K$), the performance of the PN-SBF converges toward SLNR-SBF. This is because the PN-SBF structure becomes identical to the SLNR-SBF when there is only one user group. However, except during extreme cases, the PN-SBF outperforms the conventional SBF schemes in massive MIMO systems, which verifies the effectiveness of the proposed PN-SBF under the network environment with a distinct spatial correlation.

## 6. Conclusions

In this paper, we proposed a new beamforming scheme that is called the PN-SBF for multiuser FDD massive MIMO systems with distinct spatial channel correlations when only a statistical CSI is available at the BS. From the analysis, we verified that the interference from the low-correlation user group to the high-correlation user group should be completely eliminated to maximize the sum rate of the massive MIMO systems with distinct spatial correlations. Therefore, the proposed PN-SBF applies a pre-beamforming matrix that is based on the ZF-based design principle to the low-correlation group, which eliminates the inter-group interference from the low-correlation group to the high-correlation group. In addition, to handle the intra-group interference in each group, the proposed PN-SBF additionally applies post-beamforming vectors that are designed in the manner of maximizing the SLNR to both groups. By doing this, the proposed PN-SBF effectively utilizes the spatial degrees of freedom in massive MIMO systems with distinct spatial correlations, which was verified from the simulation results.

We considered the uniform circular array as the antenna array structure for a simple modeling of spatial correlations with AS, and the proposed scheme is also applicable to other antenna array structures such as the uniform linear array and uniform planar array. Further, this study can be extended to more general spatial correlation scenarios (e.g., more than two user groups) and multi-antenna users. In addition, the joint optimization of the pre-beamforming matrix and the post-beamforming vectors can be investigated. These topics can be addressed in future works.

## Appendix A. Proof of Lemma 1

Let u denote the M×1 unit-norm vector that is isotropically distributed on a unit-radius complex sphere in *M*-dimensions. By selecting a random point on the surface of a unit sphere, it can be modeled as a normalized Gaussian random vector [46]; hence, we can write $u=x/∥x∥$ with $x∼CN\left(0,I_{M}\right)$. Therefore, when considering the law of large numbers, the distribution of u asymptotically follows a Gaussian distribution as *M* increases, which can be expressed as

(A1) $$u\approx \frac{1}{\sqrt{M}}x∼CN\left(0,\frac{1}{M}I_{M}\right).$$

Next, let us consider an M×N(M>N) random matrix U with $U^{H}U=I_{N}$, where the *i*th column vector of U, $u_{i}$ is an M×1 random unit-norm vector. By applying the Gaussian approximation, $u_{i}$ can be expressed as $u_{i}\approx \frac{1}{\sqrt{M}}x_{i}$∀i. Furthermore, by applying the law of large numbers, ${∥u_{i}∥}^{2}\overset{a.s.}{\underset{M\to \infty }{\to }}1$, ∀i and $u_{i}^{H}u_{j}\overset{a.s.}{\underset{M\to \infty }{\to }}0$∀i,j≠i. Therefore, $U^{H}U\overset{a.s.}{\underset{M\to \infty }{\to }}I_{N}$. Consequently, as *M* increases, the distribution of U can be asymptotically written as

(A2) $$\mathrm{vec}\left(U\right)∼CN\left(0,\frac{1}{M}I_{MN}\right).$$

By assuming the above Gaussian approximation, the following corollaries can be derived.

**Corollary** **A1.**
*Consider an M×M positive semi-definite matrix R and an M×1 random unit-norm vector u that is independent of R. Subsequently, the following equation holds.*

(A3) $$E_{u}\left[u^{H}\mathrm{Ru}\right]=\frac{1}{M}\sum_{i=1}^{r}\lambda_{i},$$

*where r=rank(R) and $\lambda_{i}$ is the ith eigenvalue of R.*

**Proof.** Let us define $R=V\Lambda V^{H}$ using the eigen decomposition of R, where $\Lambda =\mathrm{diag}\left(\lambda_{1},\ldots ,\lambda_{r}\right)$. Then,

$$\begin{array}{cc} E\left[{∥R^{1/2}u∥}^{2}\right] & =E\left[{∥\Lambda^{1/2}V^{H}u∥}^{2}\right]\\ & =\mathrm{tr}\left\{E\left[\Lambda^{1/2}V^{H}uu^{H}V\Lambda^{1/2}\right]\right\}\\ & =\mathrm{tr}\left\{\Lambda^{1/2}V^{H}E\left[uu^{H}\right]V\Lambda^{1/2}\right\}\\ & =\frac{1}{M}\mathrm{tr}\left\{\Lambda \right\}=\frac{1}{M}\sum_{i=1}^{r}\lambda_{i}. \end{array}$$

□

**Corollary** **A2.**
*Consider an M×M positive semi-definite matrix R and an M×N random matrix U with $U^{H}U=I_{N}$ that is independent of R. Subsequently, the following equation holds.*

(A4) $$E_{U}\left[{∥U^{H}\mathrm{RU}∥}_{F}\right]=\frac{N}{M}\sum_{i=1}^{r}\lambda_{i}.$$

*where r is rank of R and $\lambda_{i}$ is the ith eigenvalue of R.*

**Proof.** Because $R=V\Lambda V^{H}$, we have

$$\begin{array}{cc} E\left[{∥R^{1/2}U∥}_{F}^{2}\right] & =\mathrm{tr}\left\{E\left[\Lambda^{1/2}V^{H}UU^{H}V\Lambda^{1/2}\right]\right\}\\ & =\mathrm{tr}\left\{\Lambda^{1/2}V^{H}E\left[UU^{H}\right]V\Lambda^{1/2}\right\}\\ & =\mathrm{tr}\left\{\Lambda^{1/2}V^{H}\frac{1}{M}E\left[\sum_{i=1}^{N}u_{i}u_{i}^{H}\right]V\Lambda^{1/2}\right\}\\ & =\mathrm{tr}\left\{\Lambda^{1/2}V^{H}\left(\frac{N}{M}I_{M}\right)V\Lambda^{1/2}\right\}\\ & =\frac{N}{M}\sum_{i=1}^{r}\lambda_{i}. \end{array}$$

□

For the desired signal term in (21), from Corollary A2, we have

(A5) $$\begin{array}{cc} E_{E}\left[{∥{\hat{R}}_{k}∥}_{F}\right] & =E_{E}\left[{∥{\left(E_{k}^{\left(0\right)}\right)}^{H}R_{k}E_{k}^{\left(0\right)}∥}_{F}\right]\\ & =\frac{n_{k}}{M}\sum_{i=1}^{r_{k}}\lambda_{k,i}=\sum_{i=1}^{{\hat{r}}_{k}}{\hat{\lambda }}_{k,i}. \end{array}$$

Thus, we can consider ${\hat{\lambda }}_{k,1}\frac{n_{k}}{M}\lambda_{k,1}$. Therefore, we approximate the largest eigenvalue as ${\hat{\lambda }}_{k,1}\approx \frac{n_{k}}{M}\lambda_{k,1}$.

Meanwhile, for the multiuser interference term in (21), the expectation in the terms of w can be obtained from Corollary A1 as

(A6) $$E_{w}\left[w_{j}^{H}{\left(U_{k}^{\circ }\right)}^{H}\Lambda_{k}^{\circ }U_{k}^{\circ }w_{j}\right]=\frac{1}{M}\sum_{i=r_{k}^{*}+1}^{r_{k}}\lambda_{k,i}.$$

## Appendix B. Proof of Theorem 1

The upper bound of the expected statistical SINR in (23) for a high ρ is given by

(A7) $${\bar{\mathrm{SINR}}}_{k}^{\mathrm{upper}}\overset{a.s.}{\underset{\rho \to \infty }{\to }}\frac{\frac{M-r_{l}^{*}}{M}\lambda_{k,1}}{\frac{M-r_{k}^{*}}{M}\lambda_{k,r_{k}}}=\frac{n_{k}}{n_{l}}\kappa \left(R_{k}\right),$$

where $\kappa \left(R_{k}\right)=\frac{\lambda_{k,1}}{\lambda_{k,r_{k}}}$ by the definition of the condition number, $n_{k}=M-r_{l}^{*}$, and $n_{l}=M-r_{k}^{*}$. Hence, the upper bound of the sum rate can be expressed by

(A8) $$\begin{array}{cc} R_{\mathrm{sum}}^{\mathrm{upper}} & =\log_{2}\left(1+\frac{n_{k}}{n_{l}}\kappa \left(R_{k}\right)\right)+\log_{2}\left(1+\frac{n_{l}}{n_{k}}\kappa \left(R_{l}\right)\right)\\ & =\log_{2}\left(1+\frac{n_{k}}{n_{l}}\kappa \left(R_{k}\right)+\frac{n_{l}}{n_{k}}\kappa \left(R_{l}\right)+\kappa \left(R_{k}\right)\cdot \kappa \left(R_{l}\right)\right). \end{array}$$

Because $\kappa \left(R_{k}\right)\cdot \kappa \left(R_{l}\right)$ in (A8) is a constant value, the optimization problem to maximize $R_{\mathrm{sum}}^{\mathrm{upper}}$ is simply formulated as

(A9) $$\left(n_{k,\mathrm{opt}},n_{l,\mathrm{opt}}\right)=\underset{\left(n_{k},n_{l}\right)\in \Omega }{\arg\max}\left(\frac{n_{k}}{n_{l}}\kappa \left(R_{k}\right)+\frac{n_{l}}{n_{k}}\kappa \left(R_{l}\right)\right),$$

where $\Omega =\left\{\left.n_{i}\right|n_{i,\min}\leq n_{i}\leq M,\;i=k,l\right\}$ is a feasible region, $n_{k,\min}=M-r_{l}$, and $n_{l,\min}=M-r_{k}$.

Although the objective function of (A9) is not convex, it can be shown that the optimal solution is always on the boundaries of Ω by using the monotonicity of the objective function. Using the parameter $t=\frac{n_{k}}{n_{l}}$, the optimization problem in (A9) can be rewritten in terms of *t* as

(A10) $$t_{\mathrm{opt}}=\arg\max\left(\kappa \left(R_{k}\right)\cdot t+\kappa \left(R_{l}\right)\cdot \frac{1}{t}\right),$$

where $\Omega^{\prime }=\left\{\left.t\right|\frac{n_{k,\min}}{M}\leq t\leq \frac{M}{n_{l,\min}}\right\}$.

Therefore, for the case of $\kappa \left(R_{k}\right)\geq \kappa \left(R_{l}\right)$, the optimization problem in (A10) can be resolved as described below.

- *(Case i)* When $1\leq t\leq \frac{M}{n_{l,\min}}$, f(t) is a monotonically increasing function of *t*; thus, the optimal solution is $t_{\mathrm{opt}}=\frac{M}{n_{l,\min}}$.
- *(Case ii)* When $\frac{n_{k,\min}}{M}\leq t<1$ and $\frac{\kappa \left(R_{l}\right)}{\kappa \left(R_{k}\right)}\leq \frac{n_{k,\min}}{M}$, f(t) is a monotonically increasing function of *t*; thus, the optimal solution is $t_{\mathrm{opt}}=1$.
- *(Case iii)* When $\frac{n_{k,\min}}{M}\leq t<1$ and $\frac{\kappa \left(R_{l}\right)}{\kappa \left(R_{k}\right)}>\frac{n_{k,\min}}{M}$, f(t) is a monotonically decreasing function of *t* for $\frac{\kappa \left(R_{l}\right)}{\kappa \left(R_{k}\right)}\leq t<1$ and a monotonically increasing function of *t* for $\frac{n_{k,\min}}{M}\leq t<\frac{\kappa \left(R_{l}\right)}{\kappa \left(R_{k}\right)}$. Therefore, the optimal solution $t_{\mathrm{opt}}$ is $\frac{n_{k,\min}}{M}$ or 1.

Consequently, $t_{\mathrm{opt}}$ belongs to $\frac{M}{n_{l,\min}}$, $\frac{n_{k,\min}}{M}$, and 1. From this, by defining $\delta_{1}\overset{\Delta }{=}f\left(\frac{M}{n_{l,\min}}\right)-f\left(\frac{n_{k,\min}}{M}\right)$ and $\delta_{2}\overset{\Delta }{=}f\left(\frac{M}{n_{l,\min}}\right)-f\left(1\right)$, we have

$$\begin{array}{cc} \delta_{1} & =\frac{\left(n_{k,\min}+n_{l,\min}\right)\left(aM^{2}-bn_{k,\min}n_{l,\min}\right)}{Mn_{k,\min}n_{l,\min}}\geq 0,\\ \delta_{2} & =\frac{\left(M-n_{l,\min}\right)\left(aM-bn_{l,\min}\right)}{Mn_{l,\min}}\geq 0. \end{array}$$

Therefore, for $\kappa \left(R_{k}\right)\geq \kappa \left(R_{l}\right)$, $t_{\mathrm{opt}}=\frac{M}{n_{l,\min}}$, and the optimal solution is $\left(r_{k,\mathrm{opt}}^{*},r_{l,\mathrm{opt}}^{*}\right)=\left(r_{k},0\right)$.

Equivalently, for the case of $\kappa \left(R_{k}\right)<\kappa \left(R_{l}\right)$, the optimal solution is $\left(r_{k,\mathrm{opt}}^{*},r_{l,\mathrm{opt}}^{*}\right)=\left(0,r_{l}\right)$.
